# Development of Bioactive Glass-Collagen-Hyaluronic Acid-Polycaprolactone Scaffolds for Tissue Engineering Applications

**DOI:** 10.3389/fbioe.2022.825903

**Published:** 2022-02-18

**Authors:** N. N. Zurita-Méndez, G. Carbajal-De la Torre, M. V. Flores-Merino, M. A. Espinosa-Medina

**Affiliations:** ^1^ Facultad de Ingeniería Mecánica, Universidad Michoacana de San Nicolás de Hidalgo, Morelia, México; ^2^ Facultad de Química, Universidad Autónoma del Estado de México, Toluca de Lerdo, México

**Keywords:** composite coating, scaffold, eggshell membrane, bioactive glass, electrochemical techniques, biodegradability

## Abstract

In this work, bioactive glass (BG) particles synthesized by a sol-gel method, hyaluronic acid (HYA) and collagen (COL) extracted from chicken eggshell membrane (ESM), and as-purchased polycaprolactone (PCL) were used to obtain a novel bioactive scaffold using the gel-pressing technique. Two composite mixtures in weight percent were obtained and identified as SCF-1 and SCF-2, and were characterized by using FTIR, XRD, and SEM techniques. Subsequently, the composite materials applied as coatings were evaluated in simulated body fluid solutions using electrochemical techniques. The results of bioactivity and biodegradability evaluations, carried out by immersing in simulated body fluid and phosphate-buffered saline solution, showed that the SCF-1 sample presented the best biocompatibility. In accordance with the potentiodynamic results, the 316L-SS and the SCF-1-coated SS showed a very similar corrosion potential (*E*_*corr*_), around −228 mV, and current density (*i*_*corr*_) values in close proximity, while the SCF-2-coated SS showed more positive *E*_*corr*_ around −68 mV and lower *i*_*corr*_ value in one order of magnitude. These results agree with those obtained by electrochemical impedance spectroscopy, which show a corrosion mechanism governed by activation and finite diffusion through the porous layer. In addition, results were complemented by dynamic compression testing under oscillating forces to identify the developed scaffolds’ response under external forces, where the SCF-1 scaffold presented a maximum compression. The degradation resistance, bioactivity, and mechanically obtained measurements provided interesting results for potential further studies in tissue engineering.

## 1 Introduction

One of the challenges in developing substitute materials for use in biomedical engineering applications is satisfying the requirement to develop them while meeting societal and environmental expectations and requirements. The materials used in these biomedical applications should be synthesized using methods which avoid the use of toxic solvents while promoting their compatibility and enriching and enhancing their properties as a consequence of the interfacial interaction between macromolecules and the inorganic surface. Certain factors must be considered in the selection and use of materials in biological applications, such as their mechanical behavior, thermal and electrical conductivity, biostability, biocompatibility, biodegradability (anodic dissolution rate), diffusivity, and permeability of body fluids.

As a one of the important rigid structures present in the body, human and animal bone is mainly composed of hydroxyapatite (HAp) and collagen (COL) (primarily type I, but also type II, type IV, and fibrillin), it can be classified as compact (cortical) bone with a porosity around 3–5% and trabecular bone (cancellous) with an average porosity of 50–90%. At the micron- and nano-scales, aggregated type-I collagen and HAp form the collagen fibril. Mechanical properties are dependent on age, bone quality, and type. Compact bone is stronger in compression with a Young’s modulus of 7–16 GPa. While trabecular bone, with a modulus between ∼0.05–0.5 GPa, is an anisotropic and porous composite, its mechanical properties depend not only on the porosity but also on the arrangement of each trabecula. (Wang et al., 2016; Kaczmarek et al., 2017).

Scaffold design and fabrication play an important role in tissue regeneration and repair; and in the last 3 decades, there have been reported studies on materials for scaffold materials for potential applications in tissue engineering (Dhandayuthapani et al., 2011). Bioactive glasses (BGs) have been widely used for diverse clinical applications since 1985, when L.L. Hench (Hench, 2006) developed bioactive glasses. Some examples of their application are in dental (El-Sayed et al., 2020), maxillofacial (Profeta and Huppa, 2016), and orthopedic (Marques et al., 2020) treatments and recently in the development of scaffolds for bone regeneration applications, which have delivered excellent results *in vitro*. This is potentiated by the development and use of 3D-scaffolds with a similar microstructure and interconnected porosity, in order to obtain the same mechanical properties as those of cancellous bone (Bellucci et al., 2011; El-Rashidy et al., 2017). However, the application of bioactive glass scaffolds for the repair of load-bearing bone defects is limited by their low mechanical strength, and fracture toughness (Fu et al., 2011). Therefore, biodegradable polymers have been used in conjunction with bioactive glass as composite materials. Subsequently, polycaprolactone (which is a biocompatible and biodegradable polyester with high availability and suitability for manufacture) has been applied in bone scaffolds as reinforcement because of its physical, biological, and mechanical properties (Cannillo et al., 2010; Jinga et al., 2020).

Hyaluronic acid (HYA) is another important component of the extracellular matrix in the human body and has also been used in bone regeneration due to its function as carrier for bioactive components, showing excellent potential for improving osteogenesis and mineralization (Collins and Birkinshaw, 2013; Chang et al., 2016; Zhai et al., 2020). Also, the collagen increases the osteoblast differentiation (Santos et al., 2015; Li et al., 2021) associated to its set of three polypeptide chains grouped in a helical structure. One of the natural compounds that can supply both components with HYA and COL is the hen's eggshell membrane (ESM), which was used in this work. There are several research studies that report on its application in foods, cosmetics, and biomedical applications, due to its very low autoimmune and allergic responses. ESM contains type I, type V, and type X collagen principally, and several amino acids as arginine, glutamic acid, methionine, valine, cystine, and proline (Ruff et al., 2009; Zhao and Chi, 2009; Ürgeová and Vulganová, 2016; Matsuoka et al., 2019). The addition of COL within a synthetic polymer network improves both mechanical and biological properties in biomaterial applications. Here, polycaprolactone (PCL) takes the mechanical support role in the microstructure, and the collagen provides cell recognition signals which are crucial for cell behavior and development (Dong and Lv, 2016). The assessment of PCL/BG coatings on metallic substrates and the behavior under corrosive environments as well their mineralization effects have been subsequently reported (Yazdimamaghani et al., 2015; Jinga et al., 2020), although metallic implants are currently used in orthopedics, oral, and maxillofacial surgery, and they present serious disadvantages because of the release of metallic ions into the body. Therefore, to minimize this anodic dissolution, the assessment surface modification of these metallic substrates have been studied by techniques such as dip coating, deposition by spraying, and electrospinning, among others (Panahi et al., 2020).

The aim of this research was to develop porous scaffolds with potential applications in bone tissue regeneration, with a focus on improvement in mechanical properties, biocompatibility, ion diffusion, as well as fluid flux permeation to allow the nucleation and regrowth of bone phase by the use of compatible biomaterials and natural sources of collagen. No reports of scaffolds with these characteristics have been found in the literature to date, thus the addition of ESM in these scaffolds involves a new and safe possibility to incorporate an extracellular matrix formed with collagen-like proteins that could regulate calcitic biomineralization (Shi et al., 2021), allowing its potential application as a novel biomaterial in tissue engineering applications. In order to achieve the aims of this study, the microstructure and morphology of the obtained scaffolds were characterized by FTIR, XRD, and SEM. The mechanical compression properties of the scaffolds were obtained with a size relationship length-diameter of 2:1. In addition, the electrochemical evaluations in the SBF solution at 37°C ± 1°C of the composite materials applied as coatings were performed using electrochemical techniques of direct and alternating current.

## 2 Materials and Methods

### 2.1 Bioactive Glass Synthesis

Reactive-grade precursors were used to obtain BG without further purification. The BG was synthetized by the sol-gel method, through hydrolysis, condensation, and thermal decomposition of the precursors forming a stable solution until the formation of a net structure, which was then aged and thermally treated. For the preparation of the BG, the following reactants were used: tetraethyl silicate (TEOS) 98% (Sigma-Aldrich^®^), 0.1 M nitric acid (HNO_3_) (JT Baker^®^), triethyl phosphate (TEP) 99.8% (Sigma-Aldrich^®^), and tetrahydrate calcium nitrate (99%, Sigma-Aldrich^®^). The BG obtained was powdered and kept for later use.

### 2.2 Collagen/Hyaluronic Acid From Eggshell Membranes

Using commercially acquired eggs, the inner membranes of the eggshells were manually separated to obtain an essentially shell-free ESM. These membranes were partially hydrolyzed to eliminate the present albumen and then dried in an ARSA oven (model AR-290) for 15 days at 35°C. Once the membranes were completely dried, they were ground to a fine powder. The powder was then stored in a sealed recipient for later use.

### 2.3 BG-ESM-PCL 3D Scaffolds

The preparation of the 3D-scaffolds from the BG-ESM-PCL composites was done by first dissolving the PCL pellets (Aldrich^®^; average Mn 80,000) into chloroform (Meyer^®^; 99.8%) under continuous stirring. Then, the synthesized BG and ESM powders were added to the polymeric solution, stirring until a homogeneous phase was observed. In this work, the porosity of the scaffolds was obtained by applying the solvent casting and particle leaching method, using NaCl (60 wt. %) grains with a maximum diameter size of 500 µm, and then it was pressed into Teflon molds of 0.6 cm diameter by 1.2 cm high. The obtained 3D scaffolds were dismounted and immersed into distilled water to eliminate the NaCl, and dried in an oven.

The porosity of the scaffolds was estimated by gravimetry *via* the immersion method. This technique determines porosity by saturating the samples with ethanol at 20°C which has a density of 0.789 g/ml, and the pore volume was estimated with the weight change between the wet and dry samples. The total volume of the sample was determined by immersion in the fluid by applying the Archimedes’ principle. The porosity of the scaffolds was measured in triplicate, and the average obtained by the Eq. 1. Internal volume (*Vi*) was calculated by the saturation the scaffolds in ethanol, determining the pore volume by weight gain of the samples, then the total volume (*Vt*) was determined by measuring the change in weight of a pycnometer when filled with the scaffolds and the saturating liquid.

$$\varphi =\frac{Vi}{Vt}$$ (1)

### 2.4 BG-ESM-PCL Coatings

Coatings were applied on 316 L stainless steel (SS) pieces, with approximately 1 cm^2^ of area and 0.2 mm in thickness. The sheet pieces were sequentially polished using sandpapers of 230, 300, 500, 600, and 1,000 grades. Then, samples were chemically treated by immersing during 24 h in 6 M NaOH (Sigma-Aldrich^®^) solution at 90°C, washed with deionized water and acetone, and dried with hot air. The liquid composite was prepared by dissolving 5 g of each of the composition powders in 20 ml of reagent grade acetone (Meyer^®^), stirring continuously to obtain a homogenous mixture. The coatings on the pre-treated SS samples were applied by immersion and dip-coating technique with a rate of 176 mm/min during 30 s on the metallic samples. The thickness of the coatings applied to the substrates was measured by scanning electron microscopy (SEM), where their average thickness was estimated at approximately 1.43 µm. The corrosion behavior of the obtained coatings was evaluated by electrochemical techniques.

### 2.5 Physicochemical Characterizations

The phase identification of the crystalline products was carried out using an X-ray diffractometer (D8 Advanced Da Vinci). Scans were taken with a 2θ step size of 0.04° from 20° to 90° and counting time of 0.3 s using Cu Kα radiation. Additionally, the microstructure of the samples was examined by a high-resolution (1 nm) SEM (Jeol JSM IT300) equipped with an energy dispersive spectrometer (EDS: Bruker).

The analysis by FTIR was performed with a Bruker spectrometer model Tensor 27. The instrumental parameters assigned to the measurement of samples and background were from a 4,000–650-cm^−1^ measurement range, resolution of 4 cm^−1^, and sample scan time and background scan time of 32 scans (units cm^−1^ wave number). The assessment of the mechanical compressive properties of the composite tablets was done using a UniVert^®^ Mechanical Test System.

### 2.6 Biodegradation and Electrochemical Studies

The simulated body fluid (SBF) was prepared according to the Kokubo’s methodology (Kokubo and Takadama, 2006) with a pH of 7.46. The phosphate saline solution (PBS) was prepared by dissolving phosphate buffered saline (Sigma^®^) in 200 ml of deionized water, adjusting the solution pH in 7.43 at 25°C. The electrochemical tests were done using a potentiostat/galvanostat Gill-AC (ACM Instruments). A triple-electrode cell arrangement was used with an Ag/AgCl saturated reference electrode (SSCE), a platinum wire as auxiliary electrode (AE), and the coating samples as the working electrode (WE) with 1 cm^2^ of exposed area. Potentiodynamic measurements were carried out by applying a polarization scanning of 1 mV/s rate between the range of −500 to +1,500 mV vs. open corrosion potential (OCP). Linear polarization resistance (LPR) measurements were obtained between the polarization window of ±15 mV vs. OCP by applying a 1 mV/s scan rate every 15 min for approximately 40 h. EIS measurements were obtained in the frequency range of 30–0.01 Hz with a potential amplitude of 30 mV vs. OCP, at the start of immersion, and after approximately 40 h. Before starting the electrochemical measurements, a delay time was maintained until the OCP was stable.

## 3 Results and Discussion

### 3.1 Structural Characterization

#### 3.1.1 X-Ray Diffraction Analysis

**FIGURE 1:**
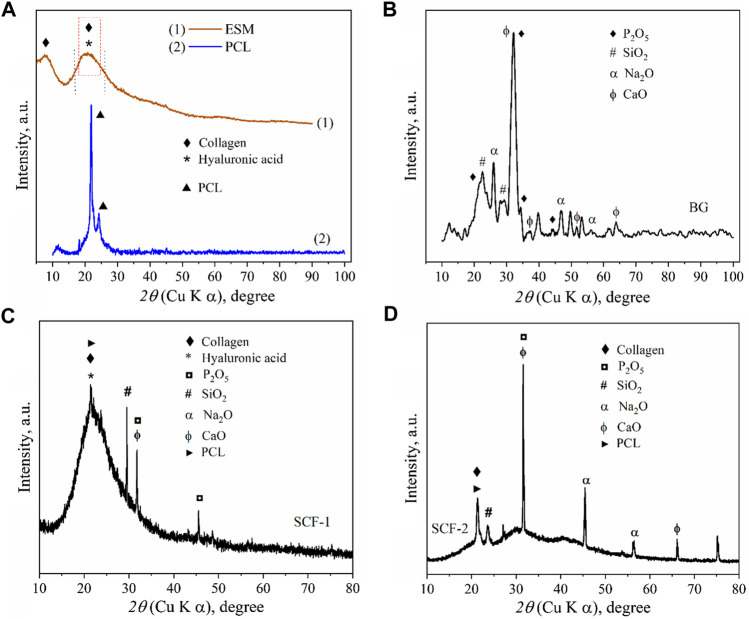
XRD patterns of: eggshell membranes conformed by collagen and hyaluronic acid and polycaprolactone **(A)**, Bioactive glass **(B)**, SCF-1 scaffold **(C)**, and SCF-2 scaffold **(D)**.

The x-ray Diffraction (XRD) pattern of the ESM sample (Figure 1A) shows the characteristic peaks of collagen with a weak diffraction peak at around 8° and a broad hump between 14° and 30°, since the collagen sample has low crystallinity (Sun et al., 2018), and the phase of hyaluronic acid is amorphous with no crystalline peaks but with a broad peak between 10° and 30° (Jia et al., 2014). This is associated with the eggshell membrane composition, which is composed principally of collagen types I and X and hyaluronic acid (Shen, 2005).

The XRD pattern of the BG samples (Figure 1B) shows the diffraction peaks associated with the SiO_2_, Na_2_O, CaO, and P_2_O_5_ compounds in accord with the crystallographic diffraction patterns PDF-00-101-0921, PDF-00-101-0876, PDF-00-100-0044, and PDF-00-154-4347, respectively. The presence of the di-phosphorous pentoxide in the bioactive glass is very desirable due to its strong stimulation in the crystallization processes, and as promoter of a further formation of the crystalline calcium phosphate layer, which forms a biomimetic hydroxyapatite composite (Cacciotti et al., 2012; Vichery and Nedelec, 2016). These results show the effect of these precursors in hydroxyapatite growth. The x-ray diffractogram in Figure 1A shows the corresponding diffraction peaks of the high purity of PCL, which was used as a polymeric matrix in the composite scaffolds.

The XRD results for the composites SCF-1 and SCF-2 show a clear difference between both samples, where the crystallinity was associated with the amount of the BG phase present in the scaffolds, as observed in Figure 1D, for the SCF-2 sample. The overlap of the peaks at around 21° forming a wide peak (Figure 1C) was associated with the signals for the polymeric phase of polycaprolactone and the collagen/hyaluronic acid contained in the eggshell membranes. Also, the components of the BG as phosphorus, silica, sodium, and calcium oxides were identified.

#### 3.1.2 Fourier Transform Infrared Spectroscopy

**FIGURE 2:**
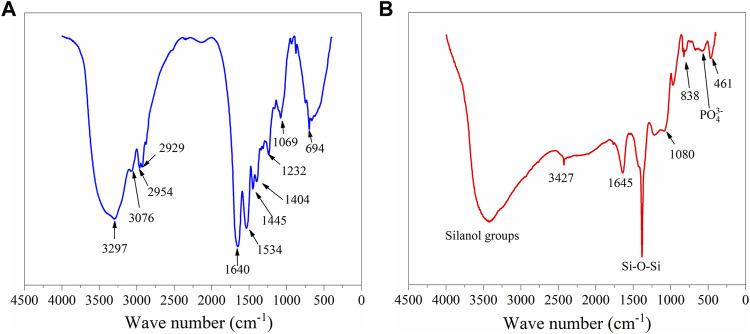
FTIR spectroscopies of the samples ESM **(A)** and BG **(B)**.

**FIGURE 3:**
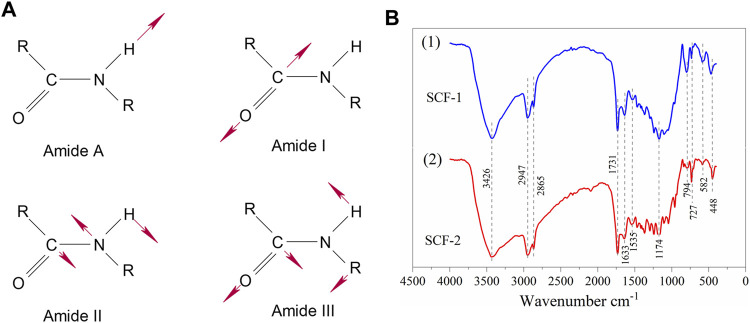
Vibrational bands of amides **(A)**. FTIR spectra for the scaffolds SCF-1 and SCF-2 **(B)**.

With IR, different functional groups absorb heat at different frequencies; the interpretation of FTIR spectra allows the identification and assessment of the conformational molecules of the materials. Figure 2A shows the FTIR spectra of ESM sample, where the polypeptides and protein units of the collagen were identified and associated to the vibrational bands as amide A and amides I–VII (Figure 3A). The wide peak observed at 3,297 cm^−1^ was related to an N-H stretching of amide A, the 3,076 cm^−1^ signal corresponds to the vibrational stretching of C-N, and the two peaks at 2,954 and 2,929 cm^−1^ were associated with the asymmetrical stretching of the CH_2_ bonds. The spectral region observed at 1,640 cm^−1^ corresponds to the protein secondary structural components of the amide I, which is due to the C=O stretching vibrations of the peptide linkages. The peak observed at 1,534 cm^−1^ is representative of amide II showing N-H deformations and C-N vibrations, and the C=H stretching located at 1,445 cm^−1^ represents the triple helical structure of collagen conformed by the three amino acids: glycine, proline, and hydroxyproline (Adochitei and Drochioiu, 2011; Foggia et al., 2011). Resulting from coupled C-N stretching and N-H bending motions, the amide III representation was observed at 1,232 cm^−1^; and out of the N-H plane, the characteristic peaks of both amide and amine vibrations were observed at 694 cm^−1^. Additionally, the hyaluronic acid phase in ESM was associated with the 1,640 cm^−1^ peak corresponding to the carbonyl group of the amide; also, the stretching of the acidic group COO^−^ and the linkage stretching of C-OH group were identified with the peaks at 1,404 and 1,069 cm^−1^ bonds, respectively.

The FTIR spectrum of BG (Figure 2B) shows the absorption peaks at 1, 386, 838, and 461 cm^−1^, associated with the bending and stretching vibrations of Si-O-Si bonds. The vibrational band with a low intensity observed at 566 cm^−1^ corresponds to the bending vibrations of the phosphate (PO_4_^3−^) groups (El-Badry et al., 2000). This suggests that phosphate can be considered as a network former (Chen et al., 2014). The wide band present at 3,427 cm^−1^ was associated with vibrations of different -OH groups, corresponding to the surface silanol functional groups of the different hydroxyl groups. These represent the superposition of the stretching modes of non-hydrogen-bonded silanols (isolated silanol groups) and the hydrogen-bonded silanol (vicinal silanol groups) (Radev et al., 2013).

The vibrational bands of the FTIR results for both SCF-1 and SCF-2 samples are shown in Figure 3B (Plot 1 and 2). The FTIR results of Figure 3B show the bond vibrations related to the hydroxyl groups at 3,426 cm^−1^ for both scaffolds. The stretching vibrations of Si-O-Si bonds were observed at 448 cm^−1^ for both SCF-1 and SCF-2, as well between the regions of 1,450–1,230 cm^−1^ with higher intensity vibrations of the stretching vibrations bonds of the Si-O-Si group (Figure 3B plot 2). The asymmetrical stretching of the CH_2_ bonds of the eggshell membrane’s collagen was identified by the peak at 2,947 cm^−1^, while, at 1,633 cm^−1^, the spectral region to the protein’s secondary structural components corresponding to amide I was observed, which is associated with to the C=O stretch vibrations of the peptide linkages. The PCL matrix was represented with the peaks at 2,947 and 2,865 cm^−1^ for the asymmetric and symmetric CH_2_ stretching, and the carbonyl group’s stretching appeared at 1731 cm^−1^. Although the FTIR spectra of composites were similar, a region between 1,245 and 856 cm^−1^ shows the difference between them because the quantity of BG in SCF-2 (60 wt. % BG) increases the intensity of its characteristic peak. Those spectra established the basis for the analysis of the biodegradation and biocompatibility of the composite scaffolds in SBF and PBS solutions when observing the adequate integration and behavior of fluids in the porous matrix.

#### 3.1.3 Scanning Electron Microscopy Analysis

**FIGURE 4:**
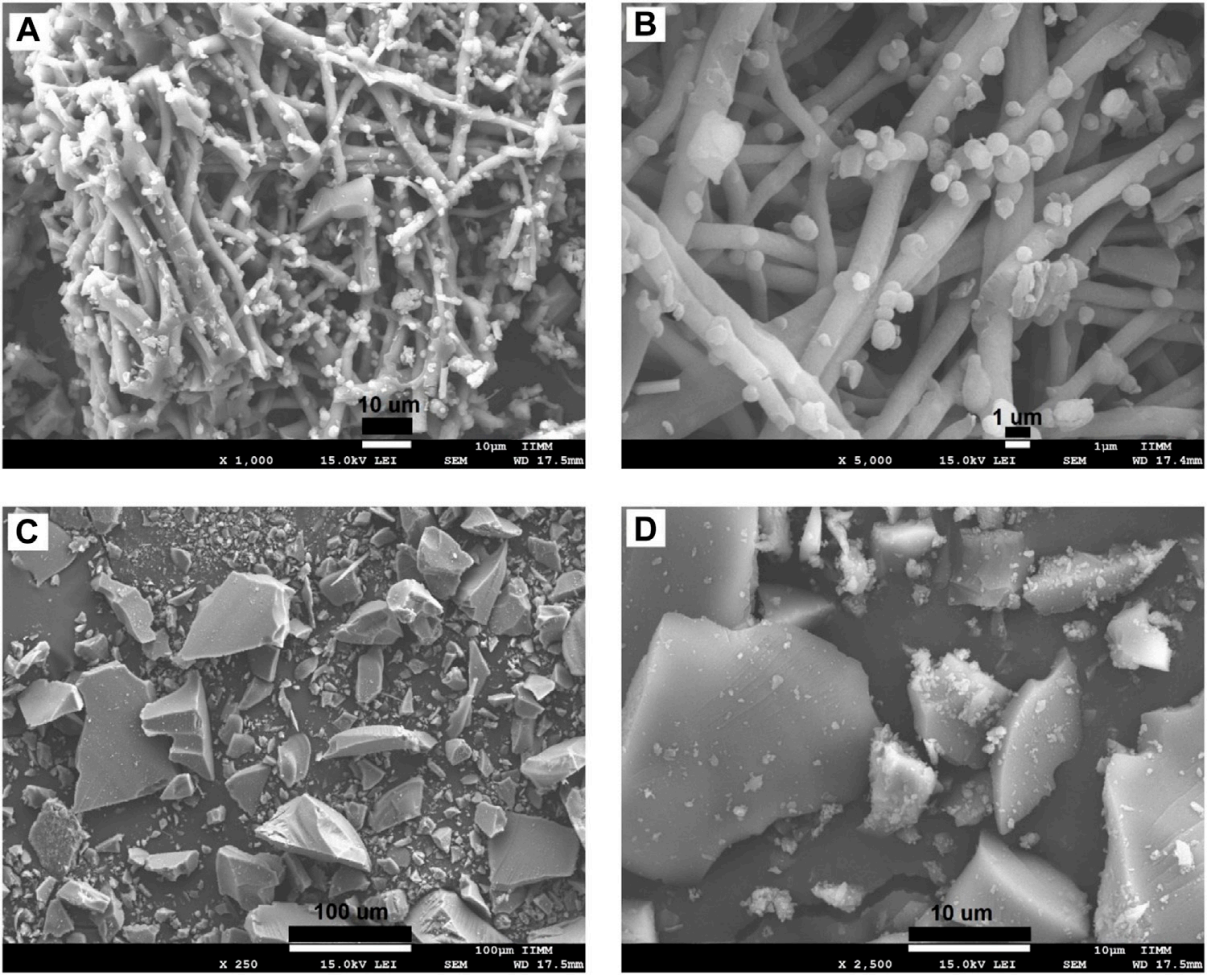
SEM microphotographs for the eggshell membranes at the magnification of: x1,000 **(A)**, x2,500 **(B)**, and BG synthetized by sol-gel technique at x250 **(C)**, and x2,500 **(D)**.

The morphologies of the ESM, BG, SCF-1, and SCF-2 observed by scanning electron microscopy (SEM) are shown in Figures 4A,B. The ESM phase displays a morphology of protein fiber meshwork highly cross-linked. This characteristic improves the structural support upon biomineralization which occurs in the egg. Ahmed et al. (2017) described the fibers of the ESM to be composed mainly of proteins (80–85%) and that the content of these proteins is predominantly collagen in its I, V, and X types. The fiber diameters were around 1–3 μm, and the presence of macropores is evident. Therefore, through this microstructure, the infiltration of precursors can occur; thus, the ESM network can be covered by the polymer phase. The morphologies of the BG (Figures 4C,D) presented irregular particle forms with a wide size distribution, from 10 µm to <50 µm. However, the surface of the BG particles was homogeneous, with a similar morphological microstructure. Table 1, shows the EDS analysis of the ESM sample. The sulfur content corresponds to the multiple disulfide-rich proteins (∼10% cysteine) that are extensively coupled by irreversible lysine-derived crosslinks, while the presence of potassium and chloride are associated to the mineral salts contained, which provides hardness, rigidity, and compression strength to the extracellular matrix of the ESM phase. Meanwhile, elemental chemical analysis of the BG indicated the presence of C, O, Si, P, and Ca.

**TABLE 1:** Chemical analysis by EDS of ESM, BG, and scaffolds samples.

ESM		BG		Scaffolds (at. %)		
Element	At. %	Element	At. %	Element	SCF-1	SCF-2
*C*	57.23	C	25.36	C	56.93	21.94
*N*	9.76	O	46.65	O	33.73	50.67
*O*	27.86	Si	25.37	Si	4.87	20.11
*S*	3.39	P	1.38	P	0.36	1.08
*Cl*	1.24	Ca	1.24	S	1.89	—
*K*	0.52	—	—	Cl	1.65	4.83
—	—	—	—	Ca	0.57	1.36

**FIGURE 5:**
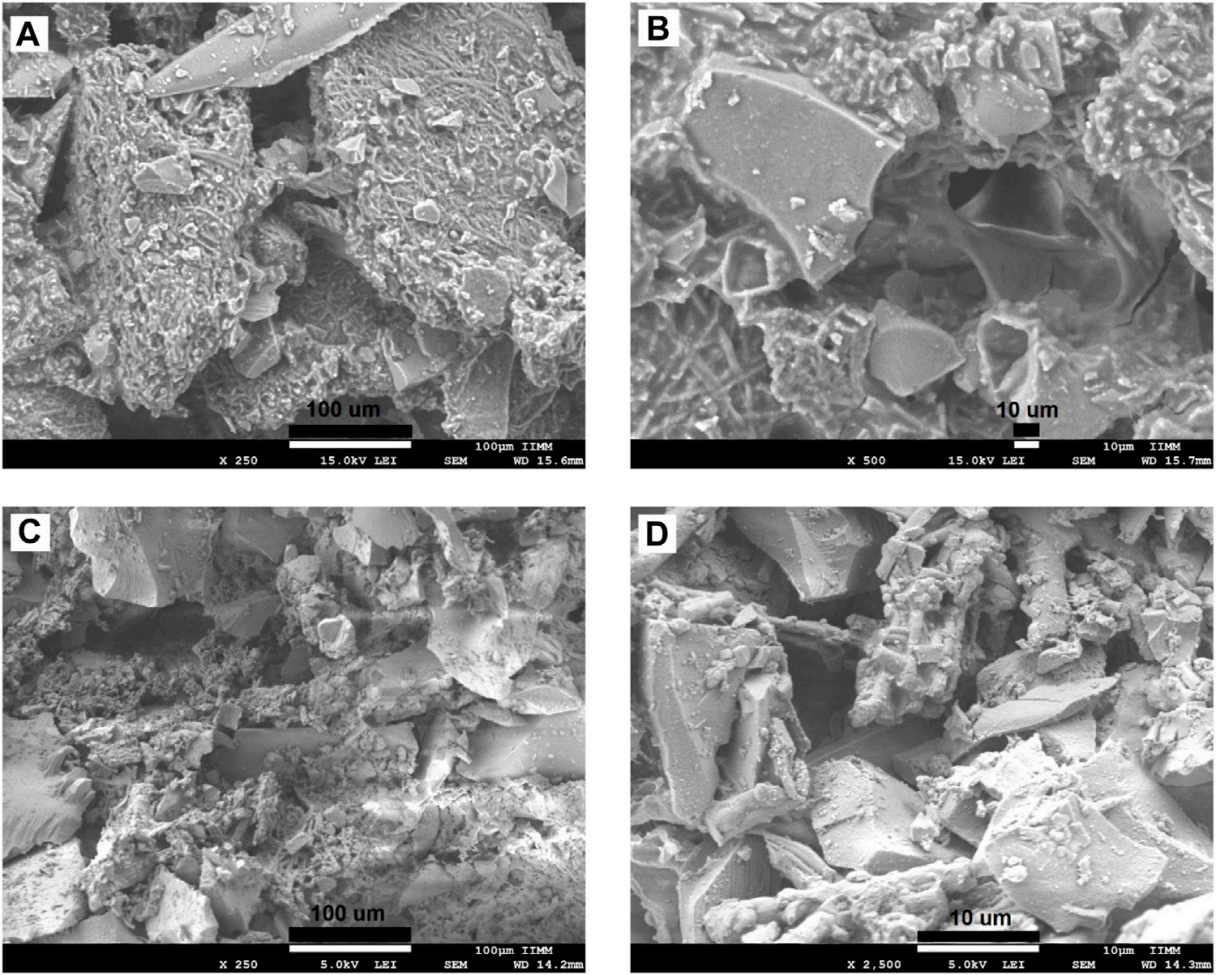
SEM images of the morphology of the SCF-1 at the magnifications of: x250 **(A)**, x500 **(B)**, and SCF-2 at x250 **(C)** and x2,500 **(D)** magnifications.

The morphologies of SCF-1 and SCF-2 are shown in Figure 5. The SCF-1 showed the BG particle surface covered by the ESM phase and homogeneously distributed in the PCL phase. The induced porosity in the scaffolds was also observed (Figure 5B), where interconnected voids of about 30 µm in diameter are present. The elemental composition of this scaffold is described in Table 1. Thus, the chemical composition was associated to a modifier effect of morphology and microstructure, as was observed in Figure 5C, for SCF-2 sample.

The morphology of SCF-2 sample shows heterogeneous particle morphology, very similar to the BG phase. The surface is significantly porous, and although the addition of the collagen phase to the BG is not pronounced (Figure 5D), it shows the proteinic fibers agglutinated by the PCL phase. The EDS analysis (Table 1) shows the reduction of the ESM content and the sulfur was not observed in this composite; in contrast, the silicon content increases proportionally with the levels of BG. Additionally, the average porosity calculated with Eq. 1 was of 0.14 and 0.28 for the scaffolds SCF-1 and SCF-2, respectively.

### 3.2 Mechanical Properties

**FIGURE 6:**
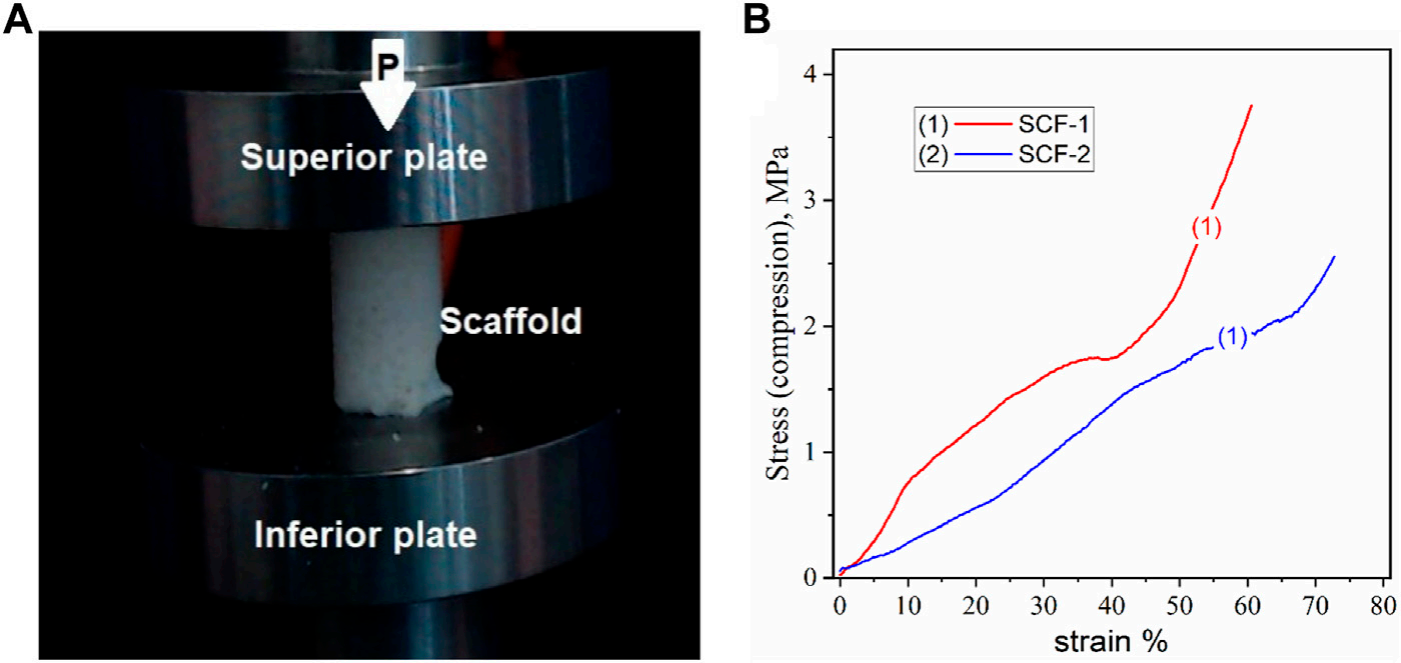
Compression test for the cylindrical porous 3D scaffolds, a) image of the sample **(A)** and the stress-strain curves **(B)**.

Cancellous bone plays an important role as the primary load-carrying and energy-absorbing component (Endo et al., 2016). It is an anisotropic and porous composite and its mechanical properties depend not only on the porosity but also on the arrangement of each trabecula, and with an elastic modulus of between 0.05 and 0.5 GPa (Wang et al., 2016; Kaczmarek et al., 2017). Trabecular or spongy bone has been demonstrated to have a greater resistance to compression than any other type of load (Careiro et al., 2013). The correlation between mechanical properties and the degree of mineralization has been studied previously, observing a general trend with an increase in the modulus with mineral content (Novitskaya et al., 2011). In order to assesses the compression properties of the developed biomaterials, cylindrical samples of the 3D scaffolds were prepared, with a size relationship length-diameter of 2:1 (Figure 6A). Figure 6B shows the strain-stress curves of the compression resistance assessment of the SCF-1 (curve 1) and SCF-2 (curve 2) samples. The mechanical behavior was nearly linear, up to 80% of the deformation for both samples, indicating an elastic response of the structured scaffolds. In this sense, the scaffold composed with higher levels of BG showed a 2.56 MPa compression strength, while the composite with higher levels of collagen/hyaluronic acid showed a strength of around 3.74 MPa. The apparent modulus of elasticity was calculated from the slope of the approximate line of the stress/strain curve from the origin to the point where the material yields. From Figure 6B, the observed slopes (*m*) over the stress/strain curve fittings for SCF-1 and SCF-2 were of 5.437 and 3.225 MPa, respectively, which corresponds to the apparent modulus of elasticity for these materials. The average Young’s modulus for trabecular bone under compression tests ranges from 1 to 22.3 GPa, and for the cortical bone, it ranges from 14.7 to 34.3 GPa (Wang et al., 2016; Kaczmarek et al., 2017; Wu et al., 2018). The reported variation is a result of physiological and pathological conditions. Although the obtained elastic module values were rather lower, it was attributed to the absence of the hydroxyapatite phase in the scaffolds, which would be formed during the bone regeneration process in *in vitro* or *in vivo* conditions. This evaluation is proposed to be carried out in a later study. Furthermore, the presence of this phase should increase the resistance of the material substantially.

### 3.3 Biodegradation and Biocompatibility Behaviour

Biodegradation and biocompatibility behaviors were determined by monitoring the change in weight during the immersion of samples in the phosphate buffer solution and simulated body fluid. For this evaluation, the scaffolds were immersed in triplicate at a temperature of 37°C for 28 days. This study was performed according to the ISO 10993-13:2010 standard (ISO, 2019). The percentage of weight loss was calculated with the Eq. 2:

$$Weightloss\left(\%\right)=100\left(\frac{W_{1}-W_{2}}{W_{1}}\right)$$ (2)

where W_1_ and W_2_ are the weight of the dry composite before and after the immersion, respectively.

**FIGURE 7:**
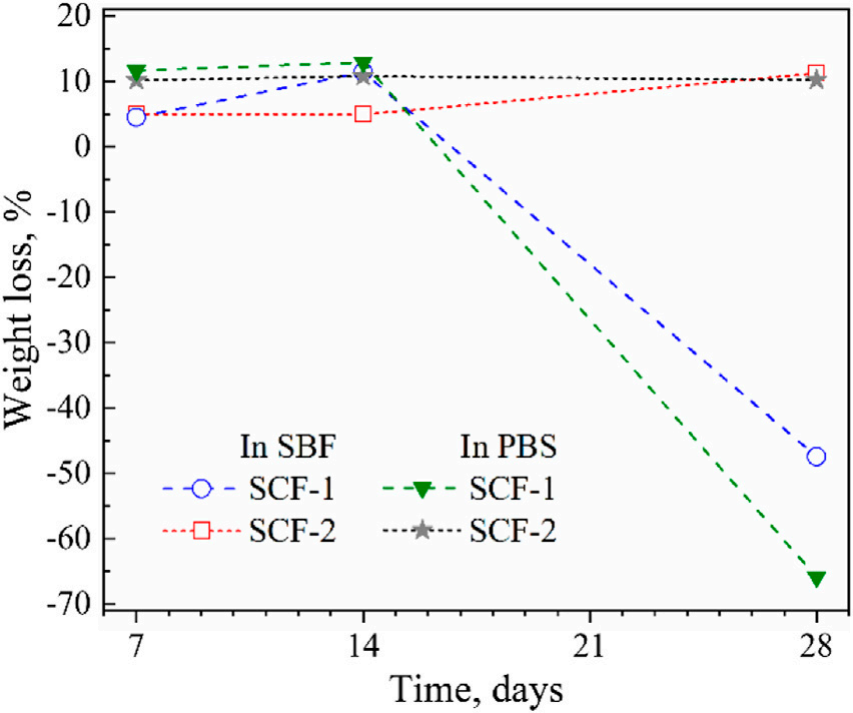
Average weight loss and gain of immersion of scaffolds into SBF and PBS.

Figure 7 shows the average weight loss rate of the composite scaffolds immersed in SBF and PBS in periods of 7, 14, and 28 days. From Eq. 2, the average weight loss remained constant during the first 14 days. For the SCF-2 (plot 2) immersed in SBF, the weight loss in the first week (7 days) was 4.93% of the initial weight. At 14 and 28 days, the observed weight loss was of 4.95% and 11.2%, respectively. This mass loss was attributed to the dissolution of the BG phase at 28 days of immersion. The behavior observed in SCF-2 scaffolds immersed in PBS showed a stable average weight loss of 10% during 28 days of immersion. This stability in biodegradation is adequate for biomaterials that are focused in osseous structures. The SCF-1 sample (plot 1) presented a linear weight loss during the first 14 days of immersion in SBF with an average weight loss of 10.5%. This percentage is slightly above those of the scaffolds with a high level of BG (which was around 5%) and indicates a larger ratio of dissolution of this material into the fluid. Furthermore, the PCL phase promoted a weight gain of 48% due to the water uptake and humidity retention of the scaffolds. The immersion of the biomaterial in PBS exhibited a behavior consistent with that observed in SBF.

**FIGURE 8:**
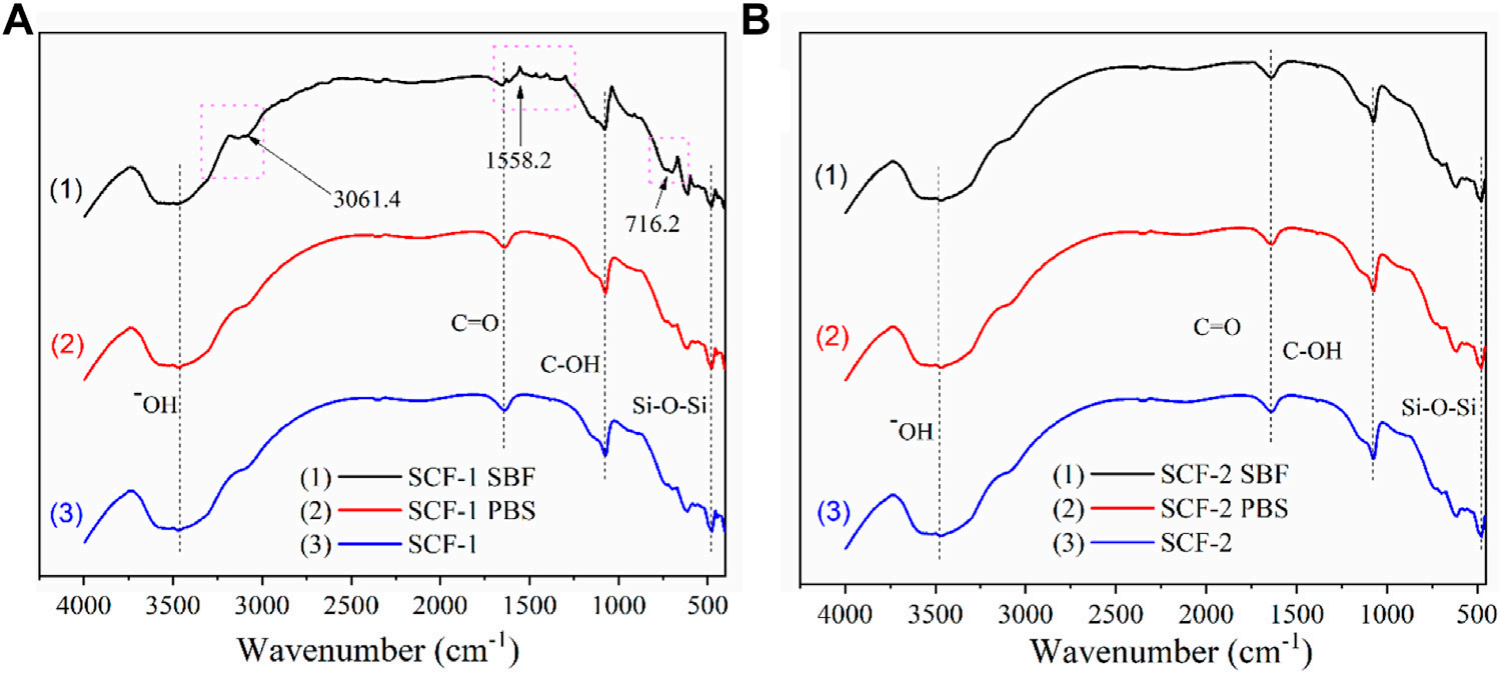
FTIR spectra for the scaffold SCF-1 **(A)** and SCF-2 **(B)** after 28 days of immersion in PBS and SBF.

The biodegradation and biocompatibility of the SCF-1 and SCF-2 samples were also evaluated by FTIR (Figure 8) in order to identify the changes in the composition of the materials after 28 days of immersion into PBS and SBF. Figure 8A shows the spectra for the SCF-1 material. As was noted, the hydroxyl groups are present at 3,462 cm^−1^ where the characteristic broad peak appears in all conditions. Both the non-immersed scaffold (SCF-1) and the PBS-immersed scaffold (curves 2 and 3) show near identical signals. This is an indicative behavior that the biodegradation during 28 days is insufficient to form new bonds in the material. Nevertheless, the SBF-immersed scaffold (plot 1) shows three different significant peaks that were related to the biocompatibility of the material in the fluid. At 3,061.4 cm^−1^, the amide B band from the collagen present in the scaffolds was observed but showed changes in the cross-linking. This was associated with the conformational change of the secondary structure of the collagen matrix. The amide I band at 1,558.2 cm^−1^ was also observed, which is mainly associated with the stretching vibrations of the carbonyl groups (C=O bond) along the polypeptide backbone. This vibration is also related to the C-N stretching and N-H bending vibrations of the conformational collagen. The major amide I band of the cross-linked BG/COLL sample is centered at 1,655 cm^−1^. In animal bone, the intensity of this band is sensitively dependent upon the extent of collagen cross-linking, which is extremely important for the mineralization process (Chang and Tanaka, 2002) in conjunction with the presence of the peak at 716.2 cm^−1^. These are representative of the phosphate band and are feasible signs of the biocompatibility of the scaffolds with the SBF media.

The FTIR spectra for SCF-2 immersed in PBS and SBF over 28 days is shown in Figure 8B. The evaluation analysis leads to the conclusion that the levels of collagen in the scaffolds improve the formation of bonds which favor the mineralization process during the immersion time. As observed in Figure 8B, the three spectra are almost identical, showing no significant differences between them. The hydroxyl groups are present at 3,507 cm^−1^, the stretching vibrations of the carbonyl groups (C=O bond) are found at 1,646.7 cm^−1^, C-OH peaks are located at 1,074.98 cm^−1^, and the presence of the Si-O-Si groups that comprise the bioactive glass phase is present at 477 cm^−1^.

FTIR results show that the BG phase added to the scaffolds presents higher dissolution in the SBF medium promoting the adequate biodegradability of SCF-2 after 28 days of immersion. Additionally, the influence of COL from ESM favors the mineralization process, which was identified by the presence of the phosphate band observed by FTIR analysis. The biocompatibility and biodegradability of both composite materials seem to be adequate in scaffolds for tissue engineering.

### 3.4 Electrochemical Evaluation

**FIGURE 9:**
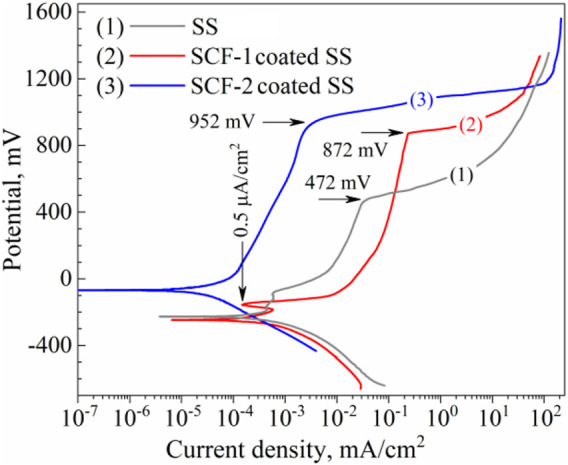
Potentiodynamic plots of the composite coatings and the substrate in SBF solution. Curves: (1) uncoated SS, (2) SCF-1-coated SS, and (3) SCF-2-coated SS, respectively.

The composites were also evaluated as coatings applied on SS substrate in SBF at 37°C ± 1°C in order to identify the corrosion mechanisms through the coating. This condition of saline solutions is representative of the behavior of the biomaterials in corporal applications. Figure 9 shows the potentiodynamic polarization curves of the BG-ESM-PCL coatings in both proportions. The SS, as well as the SCF-1-coated SS, showed a very similar corrosion potential (*E*_*corr*_), around −228 mV vs. SSCE, and current density (*i*_*corr*_) values in close proximity, of 3.59 × 10^−4^ and 1.87 × 10^−4^ mA/cm^2^, respectively, while the SCF-2-coated SS showed more positive *E*_*corr*_ and lower *i*_*corr*_ value (in one order of magnitude), −68 mV_*SSCE*_ and 2.43 × 10^−5^ mA/cm^2^, respectively. At the early polarization stages, samples showed an activation mechanism with the associated high current densities, followed by a decrease in the anodic current densities as the applied overpotential increased. These *E-i* slopes change due to a sudden increase in the current density observed above the +472, +872, and +952 mV of SS, SCF-1-coated SS, and SCF-2-coated SS, respectively, corresponding to the breakdown of limited anodic current densities, which may lead to some form of localized corrosion, such as pitting. The behaviors of the limited current density were associated with the physicochemical barrier effect of the coating (unlike for the uncoated 316L-SS, in which case, the Cr_2_O_3_ formation promotes that effect). The potentiodynamic parameters obtained from polarization plots are shown in Table 2.

**TABLE 2:** Polarization parameters obtained by the intercept method.

Sample	*i*_*corr*_ mA/cm^2^	*E*_*corr*_ mV_*SSCE*_	*β*_*a*_ mV_decade_	*β*_*c*_ mV_decade_	*E*_*transpass*_ mV
SS	3.59 × 10^−4^	−228	—	103	472
SCF-1-coated SS	1.87 × 10^−4^	−246	—	61	872
SCF-2-coated SS	2.43 × 10^−5^	−68	130	163	952

**FIGURE 10:**
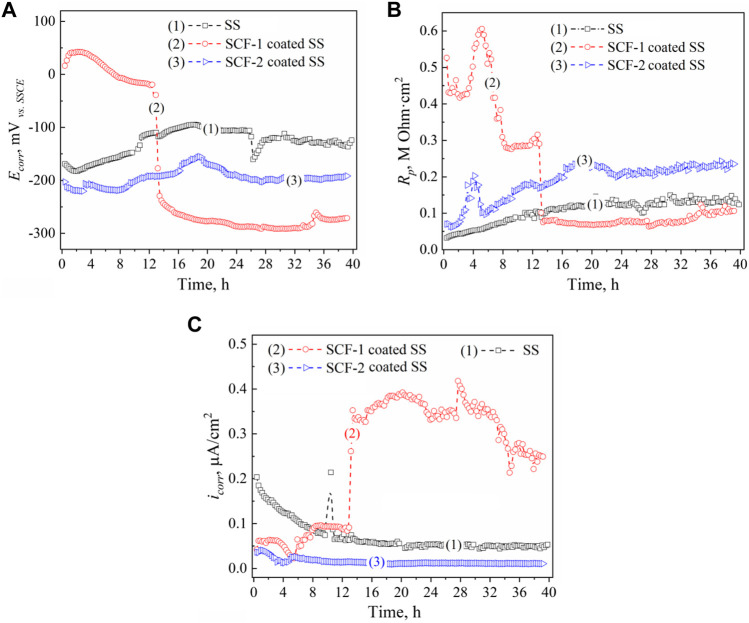
LPR results of the BG-ESM-PCL composite coatings in SBF solution: *E*_*corr*_ **(A)**, *R*_*p*_ **(B)**, and *i*_*corr*_ **(C)** kinetics. Kinetics: (1) uncoated SS, (2) SCF-1-coated SS, and (3) SCF-2-coated SS, respectively.

Results of the LPR measurements of the coatings are shown in Figure 10. The kinetics of corrosion potentials showed a slight increase in the potential for the SS and the SCF-2-coated SS but remained stable throughout the immersion time; around −120 and −200 mV_*vs SSCE*_, respectively (Figure10A). However, the SCF-1-coated SS showed its corrosion potential to be close to the SSCE reference potential during the first 12 h of immersion, later falling to about −290 mV_*vs SSCE*_ *.* Lower activity was shown by SS due to the formation of Cr_2_O_3_ as a self-protection characteristic of the alloy. The applied coatings promoted the displacement of *E*_*corr*_ to more negative values, due to the presence of other phases with a porous microstructure, and an increase in the surface area ratio of these phases and the exposed metal surface. This was more evident with the SCF-1 coating after 13 h of immersion, which showed a reduction in *R*_*p*_ associated with this increase in activity (Figure 10B). The LPR results show the more resistive behavior by the SCF-2-coated SS around 0.24 MOhm cm^2^ at the second half of immersion (*R*_*p*_ kinetics), while, the SCF-1-coated SS presented similar *R*_*p*_ values as the alloy around 0.1 MOhm cm^2^ after falling (Figure 10B).

The kinetics of the *i*_*corr*_ described by Ohm’s law was calculated using the Stern and Geary function (ASTM, 1999), with the *R*_*p*_ data and the Tafel constants (Table 2). Figure 10C shows the corrosion current behavior of the coatings. In accordance with the *R*_*p*_ results and the Ohm’s law, the SCF-1-coated SS showed the highest values of *i*_*corr*_ (0.25–0.4 μA/cm^2^ after 13 h of immersion), while the lowest values (about 0.02 μA/cm^2^) were obtained by the SCF-2-coated SS.

**FIGURE 11:**
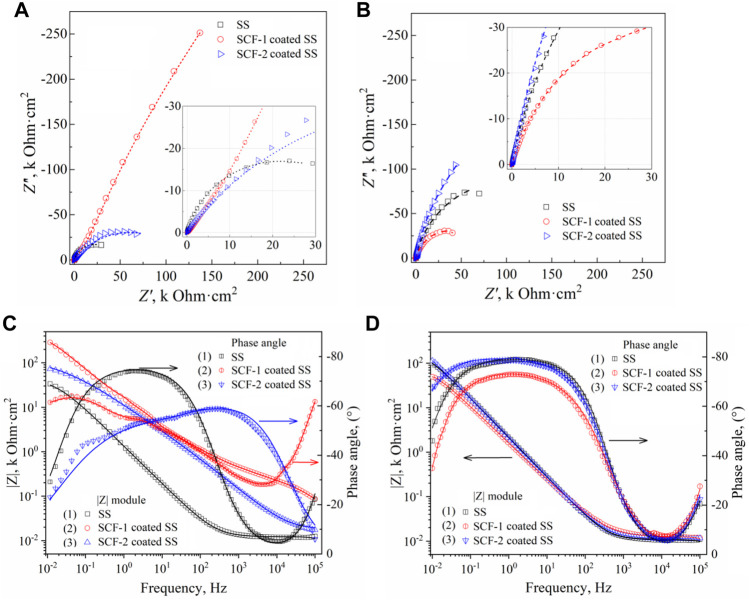
EIS results of the BG-ESM-PCL composite coatings in SBF solution. Nyquist plots at a) 1 h **(A)** and 40 h **(B)** of immersion, and Bode plots at 1 h **(C)** and 40 h **(D)** of immersion.

Electrochemical impedance spectroscopy (EIS) was used to identify the corrosion mechanisms present at the solution/coating and coating/substrate interfaces, as well as through the thickness scale. Additionally, the substrate alloy was evaluated in order to establish a baseline or reference curve. Figure 11 shows the EIS results of the coatings and the substrate at 1 h and 40 h of immersion through the Nyquist (Figures 11A,B) and Bode (Figures 11C,D) plots. The Nyquist plots obtained at both immersion times 1 h and 40 h, show the characteristic mechanisms associated with resistive behavior for the uncovered and the two coated samples. The impedance module (|*Z*|) for the SS, SCF-1-coated SS, and SCF-2-coated SS at the first hour of immersion was 33.6, 286.4, and 73.3 kOhm cm^2^, respectively (Figure 11C), and after 40 h of immersion, 100.6, 49.2, and 113.3 kOhm cm^2^ (Figure 11D). Thus, the response of SS as a substrate in the SBF solution is shown with an increase in |*Z*| magnitude of around 300 and 64% for the SS and SCF-2-coated SS, respectively, although the SCF-2-coated SS presented a substantial decrease in this parameter down to 17% of the initial impedance.

The Nyquist curves of the substrate and the composite coatings displayed a mixed mechanism of activation at the highest frequencies, while in the middle to lowest frequencies, the capacitive and finite diffusion mechanisms were observed. This was associated with ion diffusion through the physical barrier composed by the Cr_2_O_3_ self-formed film on the alloy surface and through the alloy/BG-ESM-PCL interphases of the coatings. Here, the effect of the coating microstructure on the corrosion mechanism was observed as an increase in the time for the ions to diffuse through the scale thickness, slowing down the activation mechanism at the metallic interface (Jokar et al., 2016; Zurita-Méndez et al., 2019). The phase angle Bode plots of the samples immersed for 40 h showed a wide range of frequencies (from 500 to 0.05 Hz, approximately), with a phase angle above 70° (Figure 11D), which corresponds to its capacitive and resistive behavior, in accordance with the *R*_*p*_ results of Figure 10B. This response could be of interest in biomedical applications as a scaffold in tissue engineering, allowing the controlled transport of mass through the porous microstructure. Similar to the observed LPR results (Figure 10B), the SCF-1-coated SS showed higher impedance values initially (Figure 11A), but this was lower after 40 h of immersion (Figure 11B), about 286.4 and 49.2 kOhmcm^2^, respectively.

**FIGURE 12:**
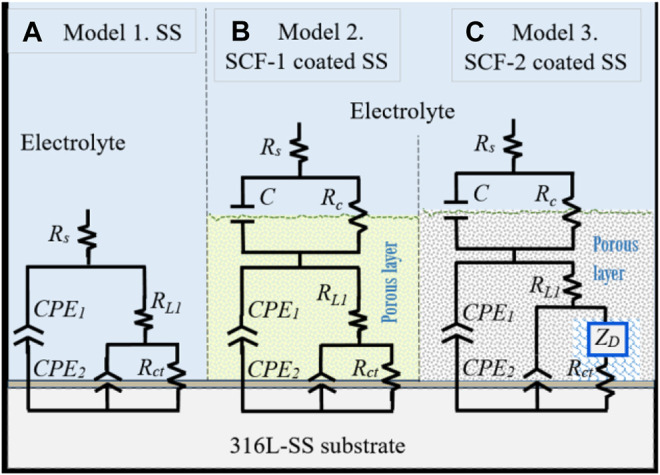
Equivalent circuit models of the corrosion mechanisms observed at the active interfaces. Model 1 for uncoated substrate, Model 2 for BG-PLA coatings. **(A)** Model 1, SS. **(B)** Model 2, SCF-1-coated SS. **(C)** Model 3, SCT-2-coated SS.

Proposed corrosion mechanisms were associated with the microstructural characteristics on the coatings composed of the mixed BG-ESM-PCL phases at two ratios, similar to an electrode with a microstructure with a superimposed porous layer (Jokar et al., 2016) that acts as a barrier against electron and ionic diffusion, thereby reducing the area for electrochemical reactions at the metallic interface (Jokar et al., 2016). However, the micro galvanic cell formation at the coating/substrate could still increase as the corrosion behavior of the substrate was associated with the activation mechanism and the diffusion through the Cr_2_O_3_ protective film. Figure 12 shows the electric circuit models (ECM) that were used as an analogy for the governing corrosion mechanism at the active surfaces and the coating thickness. For the SS, the analogic ECM corresponds to Model 1 (Figure 12A), which is composed of the electrolyte resistance (*R*_*s*_), set up in series with the parallel arrangement of a constant phase element (*CPE*_*1*_; as capacitive behavior at the double layer) and the polarization resistance (*R*_*L1*_) of the metallic surface as the activation process. The arrangement of *CPE*_*2*_, in parallel with *R*_*ct*_, represents the diffusive element manifested by the Cr_2_O_3_ protective layer inherent in the stainless steel alloys. The effect of the BG-ESM-PCL coatings is represented by a capacitance and the coating resistance in a parallel arrangement (*C*/*R*_*c*_), set in series after *R*_*s*_, Model 2 in Figure 12B. As a product of the immersion of SCF coatings during 40 h, a diffusion element (Warburg diffusion impedance; *Z*_*D*_) was included in Model 2 before *R*_*ct*_, associated with the accumulation of corrosion products on the alloy/coating interface, Model 3 (Figure 12C). As a result, a corrosion mechanism governed by transport of mass was observed.

In the ECM’s used here, the *Z*_*D*_ element (finite-length Warburg) represents the short Warburg (*W*_*s*_) element. In general, the ECM described here represents the analogue equivalent electrical circuit of the impedance for a coated electrode by a hybrid porous layer (Orazem and Tribollet, 2008). The elements of ECM are defined by the following equations:

$$Z\left(C\right)=\frac{1}{j\omega C}$$ (3)

$$Z\left(CPE_{i}\right)=\frac{1}{T_{CPE_{n}}{\left(j\omega \right)}^{\alpha }}$$ (4)

$$Z\left(R_{i}\right)=R_{S},R_{L1},\mathrm{and}R_{ct}$$ (5)

$$Z_{W_{s}}=\sigma \frac{tanh{\left(jT_{D}\omega \right)}^{P}}{{\left(jT_{D}\omega \right)}^{P}}$$ (6)

where: *R*_*s*_, *R*_*L1*_, and *R*_*ct*_ are the electrolyte resistance, the inner layer and the charge transference resistance respectively; $T_{CPE_{n}}$ is the *n* constant phase capacitance; *α* is the dimensionless potential number (0 < *α* ≤ 1; while *α* = 1 assumes *CPE* is a perfect capacitance: *C*_*dl*_). Angular frequency: *ω* = *2πf* with *f* = linear frequency, complex number $j=\sqrt{-1}$ , and *Z*_*f*_, the Faradaic impedance at the metal/scale interface. Hence, the term *T*_*D*_ represents the ratio of scale thickness *L* and the effective diffusion coefficient *D*_*eff*_ of that scale; $T_{D}=L^{2}D_{eff}^{-1}$ power is between 0 < *p* < 1, and *σ* is the constant of diffusion or the modulus of the Warburg resistance. The *CPE*_*n*_ elements (where *n = 1,2*, …) were applied instead of the perfect capacitance for better fitting. Table 3 shows the obtained values for each equivalent electric element of the ECMs used in the fitting analysis of the experimental data.

**TABLE 3:** EIS Parameters obtained by the experimental data fitting of the SS, SCF-1-coated SS, and SCF-2-coated SS samples, using Model 1 (Figure 12A), Model 2 (Figure 12B), and Model 3 (Figure 12C).

Sample	SS		SCF-1-coated SS		SCF-2-coated SS	
Immersion (h)	1	40	1^1^	40^2^	1^1^	40^2^
***R***_***s***_ (Ω·cm^2^)	2	1	0.5	0.01	16.8	0.01
***C*** (µF·cm^2^)	—	—	64.363	241.3	98.224	229.42
***R***_***c***_ (kΩ·cm^2^)	—	—	1,068.4	476.7	0.05	347.8
$T_{CPE_{1}}$ (µF·cm^2^)	282	719	28.516	438.42	26.841	261.63
***α***_***1***_ (*)	1	1	0.7896	1	0.5794	1
*R*_*L1*_ (Ω·cm^2^)	10.11	9.83	272.5	12.46	1.472	11.32
$T_{CPE_{2}}$ (µF·cm^2^)	156.4	91.89	24.727	245.99	1.646	156.79
***α***_***2***_ (*)	0.8487	0.8867	0.5759	0.7501	0.8681	0.8269
***σ*** (kΩ·cm^2^/s)	—	—	—	25.965	—	116.02
***T***_***D***_ (s)	—	—	—	67.5	—	0.28895
***P*** (*)	—	—	—	0.8622	—	0.8946
***R***_***ct***_ (k Ω·cm^2^)	43.539	206.16	461.77	11.55	121.98	0.2713

1 Model 2.

2 Model 3, (*) Dimensionless potential number of the Warburg element.

In the proposed ECMs, the *CPE*_*2*_ element was associated with the roughness of the metal surface, and the *CPE*_*1*_ with the heterogeneous microstructure of the composite coatings. For the purposes of the EIS analysis, a porous coating and homogeneous microstructure were considered, which were formed during the drying and sintering process. Although the coating surface SEM images were not presented here, the fitting Model 2 agrees with the experimental data associated with a porous microstructure. Additionally, the rugose surface of the substrate and the coating’s porosity promoted a depression of the semicircle at the high frequencies associated with an activation mechanism (for the typical Randel’s circuit). Thus, the *α*_*2*_ values (Eq. 4) are lower as shown in Table 3. Therefore, the phase angles have lower values at the higher frequencies (Figures 11C,D). From the Warburg element (Eq. 5), parameter p-values around 0.5 are associated with an infinite diffusion mechanism, although in this study, the p-values were close to 1 (Table 3), which was associated with a capacitive behavior due the resistive response of the coatings. The Bode plots showed a mixed mechanism of capacitive behavior and diffusion which caused a time delay of mass transfer of species through both the coating thickness and the Cr_2_O_3_ inner layer. After 40 h of immersion, this was observed in the loop formed between the mid to lowest frequencies (100–0.1 Hz), showing 70–80° of the phase angle (Figure 11D), as previously reported (González-Reyna et al., 2021), to which the results were a consequence of high resistance and a capacitive behavior.

The electrochemical measurement results on the composite coatings with porous microstructure characteristics, evaluated on scaffolds or coatings, showed an interesting means of mass transport and fluid permeation through the coating. This observed behavior suggests a potential application in the tissue engineering area, which could become an interesting field of study in the future. In this respect, the amide I-band centered at 1,655 cm^−1^ and its intensity was sensitively dependent upon the extent of collagen cross-linking for promoting the mineralization process (Endo et al., 2016); additionally, the presence of the peak at 716.2 cm^−1^ representative of the phosphate band is a sign of the feasible biocompatibility of the scaffolds with the SBF media. Therefore, the ionic diffusion and SBF solution permeation allow the interaction of the H_2_O molecules with the Si-O bonds in the BG network promoting the formation of Si-OH groups which attract the ions of Ca^2+^, H_2_PO_4_^−^, HPO_4_^2−^, and PO_4_^3−^ of the SBF solution favoring the phosphate composite nucleation sites. They precipitate after some immersion time; a similar observation in a hybrid composite with PCL matrix (Abdal-hay et al., 2012; Abdal-hay et al., 2013; Jokar et al., 2016), while amorphous inorganic formations of the nuclei finally crystallized into the apatite phase (Abdal-hay et al., 2012). Thus, the BG phase improvement in the formation of the HAp phase was suggested too.

The 48% weight gain shown by the SCF-1 scaffold at 28 days of immersion in the SBF solution was associated with higher permeation of fluid through the porous paths, which allowed the ion interactions with the available bonds of the composite material. Therefore, the bulk weight and ion diffusion through increases in coating thickness were observed. The lowest *R*_*p*_ values shown by this composite after 13 h of immersion (Figure 10B), as well as the lower values of the resistance elements (*R*_*c*_, *R*_*L1*_, *R*_*ct*_, and *σ*) obtained by fitting the EIS data using the Models 2 and 3 (Table 3), are correlated with the SCF-1 weight gain. This behavior was associated to the lower BG concentration on the composite (30%), while the SCF-2 showed similar weight change during the immersion time, as well as the electrochemical behavior of LPR and EIS measurements. These represent a lower mass transport rate of ions in SBF solution through the composite coating and therefore more elapsed time to get ion interaction with the composite network. However, the higher or lower elapsed time for the ion displacement could be used in specific biomedical applications, depending on the particular requirements.

## 4 Conclusion

In accordance with the results of the synthesis, characterization and evaluation of the composite materials, comprised of ESM, BG crystals, and PCL phases assessed as scaffolds or coatings, the following conclusions are described:

Microstructural characterization results showed that ESM is composed of collagen and hyaluronic acid with highly cross-linked proteinic fibers incorporated into the BG crystals and PCL matrix, forming structured morphological scaffolds. The porosity in the scaffolds was observed as interconnected voids of about 30 µm in diameter. Additionally, the XRD results for the composites SCF-1 and SCF-2 showed an increasing crystallinity associated with the amount of the BG phase present in the scaffolds.

The evaluation of mechanical properties by dynamic compression showed the scaffold composed with higher levels of BG showed minor compression strength, while SCF-1 displayed a higher compression strength related to the higher levels of collagen/hyaluronic acid present in this sample. Although, the obtained elastic module values were lower than those reported on bone assessment, they were attributed to an absence of a hydroxyapatite phase in the scaffolds.

The SCF-1 scaffold showed higher biocompatibility related to the maximum amount of collagen which started a mineralization process associated with a phosphate band observed by the FTIR analysis. Additionally, the BG phase added to the scaffolds showed a higher dissolution in the SBF medium promoting the active biodegradation of the SCF-2 after 28 days of immersion.

Potentiodynamic results showed similar values of *E*_*corr*_ and *i*_*corr*_ for SS and SCF-1-coated SS, but the *E*_*corr*_ was more positive for the SCF-2-coated SS, and the *i*_*corr*_. was significantly reduced. Additionally, the *R*_*p*_, *E*_*corr*_, and *i*_*corr*_ kinetics were similar for both SCF-2-coated SS and substrate, and SCF-1-coated SS showed the highest *R*_*p*_ values. Furthermore, the EIS results show a corrosion mechanism governed by activation and finite diffusion through the porous layer.

## Data Availability

The raw data supporting the conclusion of this article will be made available by the authors, without undue reservation.
